# Clinical characterization of dysautonomia in long COVID-19 patients

**DOI:** 10.1038/s41598-021-93546-5

**Published:** 2021-07-07

**Authors:** Nicolas Barizien, Morgan Le Guen, Stéphanie Russel, Pauline Touche, Florent Huang, Alexandre Vallée

**Affiliations:** 1 Department of Physical Medicine and Rehabilitation, Foch Hospital, Suresnes, France; 2 Department of Anesthesiology, Foch Hospital, Suresnes, France; 3 Department of Cardiology, Foch Hospital, Suresnes, France; 4 Department of Clinical Research and Innovation, Foch Hospital, 40 rue Worth, 92150 Suresnes, France

**Keywords:** Neuroscience, Physiology, Cardiology, Medical research

## Abstract

Increasing numbers of COVID-19 patients, continue to experience symptoms months after recovering from mild cases of COVID-19. Amongst these symptoms, several are related to neurological manifestations, including fatigue, anosmia, hypogeusia, headaches and hypoxia. However, the involvement of the autonomic nervous system, expressed by a dysautonomia, which can aggregate all these neurological symptoms has not been prominently reported. Here, we hypothesize that dysautonomia, could occur in secondary COVID-19 infection, also referred to as “long COVID” infection. 39 participants were included from December 2020 to January 2021 for assessment by the Department of physical medicine to enhance their physical capabilities: 12 participants with COVID-19 diagnosis and fatigue, 15 participants with COVID-19 diagnosis without fatigue and 12 control participants without COVID-19 diagnosis and without fatigue. Heart rate variability (HRV) during a change in position is commonly measured to diagnose autonomic dysregulation. In this cohort, to reflect HRV, parasympathetic/sympathetic balance was estimated using the NOL index, a multiparameter artificial intelligence-driven index calculated from extracted physiological signals by the PMD-200 pain monitoring system. Repeated-measures mixed-models testing group effect were performed to analyze NOL index changes over time between groups. A significant NOL index dissociation over time between long COVID-19 participants with fatigue and control participants was observed (*p* = 0.046). A trend towards significant NOL index dissociation over time was observed between long COVID-19 participants without fatigue and control participants (*p* = 0.109). No difference over time was observed between the two groups of long COVID-19 participants (*p* = 0.904). Long COVID-19 participants with fatigue may exhibit a dysautonomia characterized by dysregulation of the HRV, that is reflected by the NOL index measurements, compared to control participants. Dysautonomia may explain the persistent symptoms observed in long COVID-19 patients, such as fatigue and hypoxia.

Trial registration: The study was approved by the Foch IRB: IRB00012437 (Approval Number: 20-12-02) on December 16, 2020.

## Introduction

Increased numbers of COVID-19 patients, continue to experience symptoms several months after mild cases of COVID-19^1^. Reported symptoms lead to disability with debilitating fatigue, breathlessness, headaches, muscle and/or joint pain, brain fog, memory loss, sensation of pressure on the chest, palpitations, nausea, dramatic mood swings in combination with exercise intolerance and a relapsing–remitting pattern of recurrence^2^. Among these symptoms, several are related to neurological manifestations, including fatigue, anosmia, hypogeusia, and headaches^3^, and also respiratory symptoms, such as hypoxia^4^. Nevertheless, the involvement of the autonomic nervous system (ANS) which can aggregate all these neurological symptoms in COVID-19 infection has been under reported in the literature^5^. One of the last and recent hypotheses is that these symptoms could be the manifestation of a dysautonomia in long COVID-19 infection^6^.

Dysautonomia is marked by the failure or the increased of the sympathetic or parasympathetic components activities in the ANS. Dysautonomia shows numerous clinical symptoms, such as fatigue, labile blood pressure, orthostatic hypotension, dysfunction in heart rate variability (HRV), apparition of impotence, dysfunction of bladder, and damages in bowel functions. Dysautonomia could be acute or chronic, progressive but also reversible and was associated with numerous pathologies, such as alcoholism, diabetes, or either Parkinson’s disease. Dysautonomia was also associated with viral infections, including hepatitis C virus, HIV, or Epstein-Barr virus^5^. Fatigue is one of the major clinical characteristic of dysautonomia in COVID-19 patients^7–9^.

HRV measurement is a commonly used method for cardiovascular follow-up in healthy participants, athletes and cardiovascular patients^10^. HRV is mainly related to emotional, social, cognitive and health levels^11^. HRV is the main marker of the modulation of the sympathetic and parasympathetic branches of the ANS, and thus, of dysautonomia^12^. The low-frequency band reflects the modulation of sympathetic and parasympathetic branches on the heart^13^ and has been considered as an index of the sympathovagal imbalance^14^. In the current study, we apply the nociception level (NOL) index to estimate HRV and overall ANS balance. The NOL algorithm (Medasense Biometrics, Ramat Gan Israel) is a multiparameter nonlinear combination of HR, HRV, amplitude of the finger photoplethysmogram, skin conductance level, and their time derivatives, obtained using a non-invasive finger probe, displayed on the monitor screen as a scale of 0–100 derived using a random forest regression^15^.

Here, we hypothesize that dysautonomia could occur in secondary COVID-19 infection, also called “long COVID” infection. We aim to demonstrate this phenomenon by illustrating HRV dysregulation, reflected through the NOL index showing the sympathetic/parasympathetic balance, in long COVID-19 participants. We propose that the symptoms secondary to COVID-19 could be directly related to this virus or its immune-related dysregulation to the ANS, leading to transient or long-term dysautonomia.

## Methods

The present study prospectively included 39 participants from December 2020 to January 2021: 12 participants with COVID-19 diagnosis and fatigue, 15 participants with COVID-19 diagnosis without fatigue and 12 control participants without COVID-19 diagnosis and without fatigue. COVID-19 diagnosis was defined as positive polymerase chain reaction (PCR) test, serologic test or computed tomography (CT) scan. Participant’s eligibility for inclusion in the study was assessed during their long COVID-19 follow-up in the Department of physical medicine and rehabilitation of the Foch hospital (Suresnes, France). This follow-up aims to enhance the participants in physical activity through a personalized program of exercises-long COVID-19 rehabilitation. Exclusion criteria were age inferior to 18 years, Nociception level unusable, vasoactive medication, toxicological or alcoholic participants, BMI superior to 35, previous intensive care unit (ICU) hospitalization and participants legally protected or deprived of their freedom.

HRV was reflected using the NOL index, initially developed in the operating theater to guide analgesia administration during surgery. The NOL index was calculated from the extracted signals by the PMD-200 pain monitoring system. The NOL is based on a nonlinear combination of nociception-related physiologic parameters: HR, HRV (at the 0.15 to 0.4 Hz band power), amplitude of the photoplethysmography wave, skin conductance level, and their time derivatives^15^ obtained continuously using a non-invasive finger probe. The NOL is based on the advanced statistical and machine learning techniques to combine multiple signals into a single composite index. The NOL is a multiparameter nonlinear combination of HR, HRV, PPGA and SCL derived from random forest regression displayed on a scale from 0 to 100. Random forest is an algorithmic modeling approach that enables combining multiple parameters of different origin and discovering their complex nonlinear interactions^16^.

All study participants underwent the following protocol of measurements with the NOL index during their long COVID-19 follow-up consultation: step 1, five minutes lying down, step 2, five minutes standing, step 3, thirty seconds of flexion/extension and step 4, two minutes sitting down. This measure method shows many advantages as an easy and quick accessibility, a short recording time not disturbing the participant’s recovery and a lower sensitivity to breathing pattern than spectral variables^17,18^. Measures were performed each five seconds.

The study was approved by the Foch IRB: IRB00012437 (approval number: 20–12-02) on December 16, 2020. A non-opposed consent was obtained for all participants.

### Parameters

The following clinical data were collected: age, gender, weight, height, body mass index (weight (kg) divided by height (m2)), heart rate, systolic, diastolic blood pressure, oxygen saturation, comorbidities (hypertension, diabetes and respiratory diseases), loss of taste and smell, previous oxygen therapy (administrated through low-flow nasal cannula), and duration from first symptoms of COVID-19 diagnosis.

Fatigue was defined by clinicians as at least two of the following criteria: loss of body mass exceeding 10%, NIJMEGEN superior or equal to 23^19^, PCL-5 test superior to 20^20^ and 30 s of up and down test inferior to 3^21^.

### Statistical analysis

Characteristics of the study population were described as median and interquartile range (IQR) for continuous variables. Categorical variables were described as absolute numerical values and proportions. Comparisons between groups were performed using Kruskal–Wallis test for continuous variables and Chi-squared test for categorical variables. Repeated-measures mixed-models testing group effect were performed to analyze NOL index changes over time between groups. Statistics were performed using SAS software (version 9.4; SAS Institute, Carry, NC). Significance was defined by a *P* value < 0.05.

### Ethical approval

All procedures performed in studies involving human participants were in accordance with the ethical standards of the institutional and with the 1964 Helsinki declaration and its later amendments or comparable ethical standards.

### IRB approval

The study was approved by the Foch IRB: IRB00012437 (approval number: 20–12-02) on December 16, 2020. A non-opposed consent was obtained.

### Consent for publication

A non-opposed consent was obtained for all participants.

### Informed consent

Informed consent was obtained from all individual participants included in the study.

## Results

Characteristics of the participants were described in Table 1. Median average for age was 48 [12] years for patients with long COVID-19 with fatigue, 39 [10] years for patients with long COVID-19 without fatigue and 43 [9] years for the control group. Few patients of our study group presented comorbidities, only 3 patients with hypertension for long COVID-19 groups and one patient for control group, and only one patient with diabetes in long COVID-19 groups. No significant differences were observed for age (*p* = 0.085), gender (*p* = 0.594), comorbidities (hypertension (*p* = 0.688), diabetes (*p* = 0.377) and respiratory diseases (*p* = 0.224) between the three groups of the study population). 2 patients of the long COVID-19 group with fatigue and only one patient of the long COVID-19 group without fatigue were hospitalized and received oxygen therapy (*p* = 0.399). None of the participants of the study were hospitalized in ICU. 6 (54.6%) patients of long COVID-19 group with fatigue and 8 (53.3%) patients of the long COVID-19 group without fatigue presented loss of taste and smell (*p* = 0.951) (Table 1).

**Table 1:** Characteristics of the study population.

Parameters	COVID-19 with fatigue group	COVID-19 without fatigue group	Control group	*P* value
	N = 12	N = 15	N = 12	
Age (years)	48 [12]	39 [10]	43 [9]	0.085
Female	5 (41.7)	9 (60.0)	7 (58.3)	0.594
Hypertension	2 (16.7)	1 (6.7)	1 (8.3)	0.688
Diabetes	0 (0)	1 (6.7)	0 (0)	0.377
Respiratory diseases	2 (16.7)	1 (6.7)	0 (0)	0.224
Previous cardiovascular events	0 (0)	0 (0)	0 (0)	–
Previous oxygen therapy#*	2 (18.2)	1 (7.1)	–	0.399
ICU hospitalization	0 (0)	0 (0)	–	–
Neurological symptoms after COVID-19	0 (0)	0 (0)	–	–
Cardiac damages after COVID-19	0 (0)	0 (0)	–	–
Loss of taste and smell*	6 (54.6)	8 (53.3)	–	0.951
Weight before COVID-19 (kg)*	85 [31]	66 [28]	–	0.062
Weight at NoL test (kg)	85 [37]	63 [26]	80 [21]	0.101
Height (m)	1.75 [0.2]	1.66 [0.1]	1.76 [0.2]	0.499
BMI before COVID-19 (kg/m2)*	26.4 [8.6]	23.4 [6.9]	–	0.229
BMI at NoL test (kg/m2)	25.2 [8.8]	22.7 [5.9]	25.0 [7.1]	0.246
Loss of weight (median %)*	0 [9.3]	0 [9.8]	–	0.632
HR (bpm)	73 [17]	70 [11]	72 [22]	0.708
SBP (mmHg)	130 [20]	130 [33]	121 [29]	0.749
DBP (mmHg)	89 [12]	70 [19]	76 [22]	0.056
NIJMEGEN score*	36 [15]	20 [18]	–	0.002
PCL-5 score*	31 [18]	18 [19]	–	0.001
30 s of up and down test*	4 [3]	5 [2]	–	0.192
Oxygen saturation (%)	98 [3]	98 [3]	98 [2]	0.663
Duration from first symptoms of plong COVID-19 (months)*	7.5 [1.7]	7.0 [2.2]	–	0.157

BMI: body mass index.

HR: heart rate.

SBP: systolic blood pressure.

DBP: diastolic blood pressure.

ICU: intensive care unit.

*Only comparison between “covid-19 participants with fatigue and covid-19 participants without fatigue”.

^#^Oxygen therapy was administrated through low-flow nasal cannula.

Categorical values were presented as number and percentage, continuous variables were presented as median and interquartile range (IQR).

Moreover, no difference was observed duration from first symptoms of long COVID-19 between the long COVID-19 participants with fatigue and thus without fatigue (respectively 7.5 [1.7] vs 7.0 [2.2], *p* = 0.157). Long COVID-19 participants with fatigue presented higher values of NIJMEGEN and PCL-5 scores (respectively, 36 [15] vs 20 [18] *p* = 0.002 and 31 [18] vs 18 [19], *p* = 0.001) but not for loss of body mass exceeding 10% (respectively 0 [9.3] vs 0 [9.8], *p* = 0.632) and the 30 s of up and down test (respectively, 4 [3] vs 5 [2], *p* = 0.192,) than long COVID-19 participants without fatigue. No neurological symptoms were reported during COVID-19 episode, except loss of taste and smell for 14 patients, but with no difference between the two groups (*p* = 0.951). No cardiac damage were reported by the patients (Table 1).

**Figure 1:**
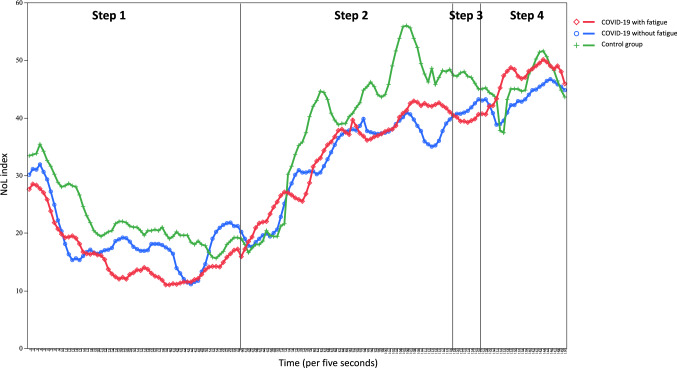
NoL index changes over time according to the group of participants. 12 participants with COVID-19 and fatigue; 15 participants with COVID-19 without fatigue, 12 control participants. *Step 1* five minutes lying down; *Step 2* five minutes standing; *Step 3* thirty seconds of flexion/extension and step 4: two minutes sitting down. Measures were performed each five seconds.

Figure 1 shows the different curves of the NOL index for long COVID-19 participants with and without fatigue compared to control participants. A significant dissociation over time between long COVID-19 participants with fatigue and control participants was observed (*p* = 0.046). A trend towards significant dissociation over time was observed between long COVID-19 without fatigue and control participants (*p* = 0.109). No difference over time was observed between the two groups of long COVID-19 participants (*p* = 0.904).

## Discussion

As our data show, possible interesting clinical aspects arise when the population is stratified between fatigue and non-fatigue long COVID-19 patients. Our population presented no significant differences in comorbidities (i.e. hypertension and diabetes, previous oxygen therapy), hemodynamic parameters (i.e. blood pressure) and duration from first symptoms. We used NIJMEGEN score and PCL-5 score to discriminate patients according to fatigue status.

We observed that HRV, reflected by the NOL index, was significant different in long COVID-19 participants with fatigue compared to control participants. In our study, the NOL index, a non-invasively measured multi-parameter index, was used to highlight the dysautonomia hypothesis in long COVID-19 participants. The variables that make up the NOL cover both sympathetic and parasympathetic activities of the ANS^22^. HRV is one of the main marker of dysautonomia^12^. HRV could be an important “window” for better understanding the inflammatory mechanism and neuroimmune system involved in long COVID-19 patients.

Recent but few experimental studies have shown the possible interest of HRV as physiological measurement parameter for COVID-19 patients^23,24^. However, this non-invasive approach to evaluate the activity of the ANS remains too little understood and requires further investigations. Recognition of dysautonomia symptomatology in long COVID-19 patients could enhance the involvement of appropriate therapy.

Some etiologies may explain dysautonomia in long COVID-19 patients including neurotropism, hypoxia, and inflammation. However, it remains unclear whether dysautonomia associated with long COVID-19 results directly from autonomic-virus pathway or post-infectious immune-mediated processes.

COVID-19 infection could be responsible for invasion of the central nervous system (CNS) by a direct hematogenous and neural propagation^25^. COVID-19 virus in the airways could pass by the epithelial barrier invading the blood and lymph circulation and thus, propagate towards the CNS. The diameter of the COVID-19 virus is 60 to 140 nm, allowing it to pass through the blood–brain barrier (BBB) and gain entry into the CNS^26^. Other hypotheses highlighted a possible polarization of neurons leading to hyposmia due to conductive loss or neural loss^27^. Possible damages to the afferent hypoxia-sensing neuros could be due to the direct effect of COVID-19 by binding ACE2 expressed in capillary endothelium of BBB to gain access into the CNS^28^ or by indirect effect with cytokine storm on mitochondria or on nerve fibers^29^. Recent PET (positron emission tomography) scan findings have highlighted that COVID-19 neurotropism could occur in patients with impairment of olfactory bulb but also in other limbic structures, including thalamus/hypothalamus, cerebellum and the brainstem^30,31^. Moreover, the neuro-invasiveness of COVID-19 could have a direct role in hypoxia^32^. Excessive accumulations of acid metabolites in brain and anaerobic metabolites in mitochondria inducing edema and obstruction of the cerebral blood flow lead to hypoxia^33^.

The glossopharyngeal afferents, which innerve the carotid body and the vagal afferents enhancing the respiratory tract, present a key action in the monitoring organelle mechanism and modulating homeostasis by the stimulation of the ANS. Theses neurons are the primary sensory inputs of numerous reflex circuits modulating several major functions, such as HR, BP, and airway caliber^34^. Pulmonary receptors expressed on afferent vagal nerve terminals in the lung have mechanical or chemical stimuli, which translocate in the brainstem by small-diameter myelinated (Aδ) or unmyelinated (C)-fiber nerve axons. C-fiber afferents respond to inflammatory markers and could have a major role in prominent action in dyspnea and breath difficulty but also a major role in coughing^34^. Vagal C-fiber afferents innervate the larynx response to laryngeal discomfort while the afferent information arrives from the vagal and glossopharyngeal nerves to merge at the nucleus of the tractus solitarius (NTS), a key site of critical homeostasis pathway. Thalamus, somatosensory cortex, amygdala and insular cortex were implicated in breath perception. However, the biological phenomenon underlying the dissociation between hypoxemia and overt dyspnea remain unknown in COVID-19 patients. Nevertheless, the observed disassociation could be present in patients presenting lesions in the glossopharyngeal or vagus nerves due to cranial nerve damages^4^. Impairs of the HRV may be explained by the acute dysautonomia enhancing by afferent baroreflex failure. This mechanism leads to several damages at the afferent baroreceptor signaling, which starts from baroreceptors in carotid bodies to the vagal and glossopharyngeal nerve fibers, and then, to the NTS^5^.

One of the aspects of COVID-19 patients’ complications is the presentation of low blood oxygenation but no sensation of dyspnea^29^. This phenomenon has been called “happy”^4,29^ or “silent”^35,36^ hypoxemia. Nevertheless, this phenomenon is baffling to physicians and explanations remain unclear and hypothetic. This suggests that at least some sensory information could reach the brainstem to elicit a partial compensatory reflex respiratory response that is sufficient to lower the CO2 level, which diffuses more rapidly across the alveoli than oxygen. Respiratory pathology involves autonomic reflexes Projections from the brainstem to the cortex allow the brain to process homeostatic afferent signaling. A signal of internal hypoxia was received by the brain to ensure a rise of sensation of “air hunger” and needing to breathe, which is curiously absent in severe COVID-19 patients^4^.

Moreover, the ANS responds quickly to changes in physiological states, it may offer signals, in the form of HRV, that can warn of an impending cytokine storm sooner than other currently employed laboratory tests^37^. COVID-19 infection was associated with a cytokine storm characterized by the release of large amounts of pro-inflammatory cytokines and chemokines^38,39^. The relationship between HRV and inflammatory factors has been well extensively studied. Inflammatory factors might across the compromised BBB and cause brain damages. Thus, inflammation caused by viral infection could trigger and propagate chronic neuronal dysregulation^40^. A recent meta-analysis demonstrated an inverse relation between HRV and inflammation^41^. The measure of HRV was inversely correlated with CRP^42^. In the current COVID-19 infection, involvement of CRP became the vital monitoring patients’ inflammatory factor^38,39^. Nevertheless, the biological mechanism for the relationship between ANS and immune system interacted to impact the HRV has not been well understood^37^.

### Limitations

The main strength of our study was the use of the NOL index, which is a validate parameter to estimate HRV^43^. The data were collected regarding standardized protocols that add validity to our study’s results. Very few studies have investigated dysautonomia in COVID-19 participants and comparison to previous literature is difficult. One major limit was the relatively small sample size limiting the statistical power of the analyses. Consequently, we cannot exclude the possibility that some of the negative findings were due to lack of statistical power. Few patients of our population were hospitalized for COVID-19 (only 3 patients received oxygen therapy), the small sample size of our population did not allowed us to investigate the aspect of the different severity of the disease and dysautonomia aspect. Main part of the study population presented mild or moderate COVID-19 disease, only 3 patients needed oxygen therapy, but no additional data for the severity of the disease was collected and can appear as a main limitation. The small sample of patients receiving oxygen therapy did not allow us to perform subgroup analyses. A main limitation of our protocol was that steps of measurements with NOL index were not randomized. Moreover, possible delayed and overlapping effects could be not exclude due to the “fast” (12 min) experimental protocol.

## Conclusion

Long COVID-19 participants with fatigue may exhibit a dysautonomia characterized by dysregulation of the HRV, reflected by the NOL index, compared to control participants. Dysautonomia may explain the persistent symptoms observed in long COVID-19 participants, such as fatigue and hypoxia. Future clinical studies are needed to validate this hypothesis, with direct measurement of HRV and with specify focus on ICU or hospitalized patients, to allow better care of these participants during long COVID-19 rehabilitation.
